# Editorial: Mucosal Vaccination: Strategies to Induce and Evaluate Mucosal Immunity

**DOI:** 10.3389/fimmu.2022.905150

**Published:** 2022-04-29

**Authors:** Pamela A. Kozlowski, Nicholas J. Mantis, Andreas Frey

**Affiliations:** ^1^ Department of Microbiology, Immunology & Parasitology, Louisiana State University Health Sciences Center, New Orleans, LA, United States; ^2^ Division of Infectious Diseases, Wadsworth Center & New York State Department of Health, Albany, NY, United States; ^3^ Division of Mucosal Immunology & Diagnostics, Program Area Chronic Lung Diseases, Research Center Borstel, Leibniz Lung Center, Borstel, Germany

**Keywords:** adjuvant, formulation, IgA, mucosal vaccine, protection, targeted delivery

## Introduction

Prevent transmission - prevent infection - prevent disease. To achieve this trifecta, a pathogen must be intercepted at the earliest stages of encounter with human tissues. Considering that the majority of infectious agents enter *via* mucosal surfaces, sterilizing immunity is difficult to achieve with a classic systemic vaccination approach. Injected vaccines almost exclusively induce humoral and cell-mediated immunity in the systemic compartment, and thus, they primarily arm the host to fight infectious agents that have already penetrated the body. To arrest pathogen entry *via* mucosal surfaces and spread to peripheral tissues, our body is able to produce and transport Immunoglobulin A (IgA) onto mucosal surfaces and to deploy pathogen-reactive B and T lymphocytes directly below. Generating these protective immune effectors requires the participation of local antigen-presenting cells, and hence, successful induction of mucosal immunity can only be achieved if the vaccine is taken up at a mucosal surface and processed in immunologically relevant mucosal lymphoid tissue. This Research Topic is intended to provide insight into the current status of mucosal vaccination approaches, the rationale for generating mucosal immune responses, and shed light on the mechanisms of protection unique to the mucosal compartments.

## Sounding the Alarm: Stimulating Immunity Through Novel Adjuvants That Mimic Pathogen-Derived Danger Signals

In this Research Topic, a number of studies investigated the use of adjuvants for improving efficacy of mucosal immunizations, and to activate the innate and adaptive immune responses therein. Richardson et al. evaluated the immune modulatory activity of Fms-like tyrosine kinase 3 ligand (FL) - a cytokine known to activate dendritic cells - as a mucosal adjuvant for a *Vibrio cholerae* ghost-vectored *Chlamydia abortus* antigen. They found that intranasal co-administration of vector with FL enhanced the number of innate cells and antigen-specific T cells in iliac lymph nodes, vaginal IgG and IgA antibodies, and protection against vaginal infection with *C. abortus* in mice. Wang et al. used ovalbumin antigen to compare the intranasal adjuvanticity of lipopolysaccharide (LPS) derived from the *Alcaligenes faecalis* commensal bacterium to the murine “gold standard” mucosal adjuvant, cholera toxin (CT). *Alcaligenes* LPS was just as effective as CT for increasing IgA antibodies in upper and lower respiratory secretions, as well as numbers of T follicular helper cells, IgA B cells and germinal centers in nasal-associated lymphoid tissue and cervical lymph nodes. Importantly, in contrast to CT, *Alcaligenes* LPS produced a more regulatory T cell response and no inflammation in the nasal cavity, a critical requirement for translation to humans.

Two papers present novel approaches for optimizing new vaccine adjuvants and formulations. Based on previous findings that certain mast cell activators can act as adjuvants, Johnson-Weaver et al. have established an *in vitro* system to screen small molecule candidates for adjuvant activity. Their cytokine profiling data demonstrates that substances which induce mast cell degranulation can stimulate innate immune cells, and subsequent *in vivo* experiments show that *in vitro* mast cell activation correlates with adjuvanticity in intranasal vaccination. In a very thorough and comprehensive analysis, Abhyankar et al. have approached the problem of optimizing an intranasal liposomal vaccine formulation containing synthetic TLR ligands to achieve maximum potency. They coupled computational modeling with an extensive immunization study where they systematically varied dose and composition of a complex vaccine formulation for the intestinal parasite *Entamoeba histolytica*. The resulting immune responses were analyzed for cellular, humoral and protective activity. Rational adjuvant design is also the topic of the study presented by Jangra et al., and their work is particularly timely in light of the questionable protection achievable with current SARS-CoV-2 vaccines. They stress the necessity for directing adaptive immune responses towards broad reactivities that confer cross-protection in the face of continuously mutating viruses and rapidly emerging pathogen variants. Importantly, intranasal delivery of a SARS-CoV-2 spike protein in their RIG-I agonist-containing nanoemulsion adjuvant elicited high titers of serum neutralizing antibodies in mice, and these antibodies passively protected naive mice against infection with a heterologous SARS CoV-2 variant.

Live attenuated pathogens or replication-incompetent bacterial/viral vectors have inherent immune stimulatory properties. Mechanistic details of such action in the lung are provided in the paper of Bhagyaraj et al. who demonstrate the activation of innate lymphoid Natural Killer cells and a link with CD8 T cell development after intranasal vaccination of mice with a mutant *Brucella* strain. Velarde de la Cruz et al. used orally administered poliovirus vectors expressing simian-human immunodeficiency (SHIV) antigens to boost mucosal immune responses in rhesus macaques orally primed with a liposome-formulated SHIV-DNA vaccine. Their data show that this vaccination regimen generated high frequencies of SHIV-specific T cells in the large intestine and the female reproductive tract, though only marginal antibody responses were attained.

A mucosal antigen-targeting approach for oral vaccination is presented by Van der Weken et al. They created a monoclonal antibody (mAb) against aminopeptidase N, a luminally-expressed membrane component of enterocytes, and show that this mAb is transcytosed by the intestinal epithelium and taken up by local antigen-presenting cells. Oral immunization of mice with this mAb coupled to a bacterial antigen elicited a specific systemic IgG response and IgA antibody-secreting cells in the mesenteric lymph nodes.

## Teaming Up - Combining Systemic and Mucosal Vaccination Regimens to Achieve Body-Wide Protective Immunity

Bosnjak et al. immunized rodents with a recombinant Modified Vaccinia Ankara virus construct expressing the SARS-CoV-2 spike protein and provide a detailed analysis of immune responses generated by different prime/boost protocols utilizing intramuscular and/or intranasal vaccinations. They obtained strong spike-specific CD8 T cell responses and neutralizing antibodies in the lung when intramuscular priming was followed by an intranasal boost, and this vaccination strategy protected hamsters against infection with SARS-CoV-2. Synergism between systemic and mucosal immunity in providing protection against rectal SHIV infection was investigated in a study by Gong et al. who demonstrated that passive immunization of rhesus macaques with an anti-HIV neutralizing antibody is more protective if systemic administration of HIV neutralizing IgG is combined with a rectally applied dimeric IgA having the same specificity. Likewise, Lakhashe et al. show that systemic priming followed by intranasal boosting with a virosomal HIV-1 gp41 vaccine in nonhuman primates can induce specific antibodies in both serum and vaginal secretions, and confer protection against low dose vaginal SHIV challenge.

## Profiteers – Mucosal Vaccination in Combatting a Swath of Infectious Agents

The articles in the Research Topic make it clear that mucosal vaccines and vaccination strategies have broad applications across viral (HIV-1, SARS-CoV-2), bacterial (*Bordetella, Escherichia, Brucella, Chlamydia*), and parasitic (*Entamoeba*) pathogens of humans and animals. Indeed, as is discussed in the review by Vaernewyck et al., mucosal vaccines may also find their way into treating common and everyday nuisances as periodontal disease. Mucosal vaccinations may even impact diverse disciplines such as aquaculture, as highlighted by Kole et al. This One Health approach to vaccination certainly has relevance in a world still reeling from the impacts of the most notorious respiratory virus in recent history: SARS CoV-2.

## Where Do We Stand? - Achievements and Gaps

For decades, the power of mucosal vaccines has been stressed, and the elimination of polio from most countries worldwide is a triumph for the global polio eradication program which relies to a large extent on the oral polio vaccine (1). Childhood mortality rates from severe gastroenteritis have also been dramatically reduced with the development of live attenuated oral vaccines for rotavirus (2).

Translating basic research into practical applications, though slow, is advancing, as exemplified by the mucosal vaccination studies against pertussis, reviewed here by Dubois & Locht. Along that line, Sánchez-Ramón et al. describe a clinical study where immunosuppressed patients who received sublingual polybacterial vaccines were significantly protected against urinary and respiratory infections. However, reducing the number of world-wide deaths from severe acute respiratory diseases is a current priority. Mucosal vaccines in development for respiratory syncytitial virus and SARS CoV-2 will hopefully fulfill this goal in the next decade. In conclusion, the collection of manuscripts presented in this Research Topic is a demonstration of the ongoing efforts in the areas of mucosal vaccination. The importance of these research endeavors is underscored when put onto the backdrop of COVID-19. Indeed, the emergence of SARS CoV-2 breakthrough infections that we have witnessed over the past year must be attributed in part to the lack of mucosal immunity elicited by existing parenteral (injected) vaccines.

## Author Contributions

All authors listed have made a substantial, direct and intellectual contribution to the work, and approved it for publication.

## Conflict of Interest

The authors declare that the research was conducted in the absence of any commercial or financial relationships that could be construed as a potential conflict of interest.

## Publisher’s Note

All claims expressed in this article are solely those of the authors and do not necessarily represent those of their affiliated organizations, or those of the publisher, the editors and the reviewers. Any product that may be evaluated in this article, or claim that may be made by its manufacturer, is not guaranteed or endorsed by the publisher.
